# Natural products target glycolysis in liver disease

**DOI:** 10.3389/fphar.2023.1242955

**Published:** 2023-08-17

**Authors:** Shenghao Li, Liyuan Hao, Xiaoyu Hu

**Affiliations:** ^1^ Chengdu University of Traditional Chinese Medicine, Chengdu, China; ^2^ Hospital of Chengdu University of Traditional Chinese Medicine, Chengdu, China

**Keywords:** glycolysis, liver disease, liver, metabolism, natural products

## Abstract

Mitochondrial dysfunction plays an important role in the occurrence and development of different liver diseases. Oxidative phosphorylation (OXPHOS) dysfunction and production of reactive oxygen species are closely related to mitochondrial dysfunction, forcing glycolysis to become the main source of energy metabolism of liver cells. Moreover, glycolysis is also enhanced to varying degrees in different liver diseases, especially in liver cancer. Therefore, targeting the glycolytic signaling pathway provides a new strategy for the treatment of non-alcoholic fatty liver disease (NAFLD) and liver fibrosis associated with liver cancer. Natural products regulate many steps of glycolysis, and targeting glycolysis with natural products is a promising cancer treatment. In this review, we have mainly illustrated the relationship between glycolysis and liver disease, natural products can work by targeting key enzymes in glycolysis and their associated proteins, so understanding how natural products regulate glycolysis can help clarify the therapeutic mechanisms these drugs use to inhibit liver disease.

## 1 Introduction

The incidence of liver disease remains increasing (Yu et al., 2014b), it ranges from simple steatosis or non-alcoholic fatty liver disease (NAFLD) or non-alcoholic steatohepatitis (NASH) to cirrhosis and liver cancer (Li et al., 2019). As the core organ of nutrient storage, synthesis and metabolism, the liver has a remarkable ability to maintain metabolic homeostasis (Petersen et al., 2017). However, under various adverse conditions, liver metabolic homeostasis is destroyed, especially glycolysis and other glucose metabolic functions. In recent years, abnormal of glycolysis has received increasing attention due to its interaction with liver disease.

Glycolysis refers to the process in which glucose is catalyzed to pyruvate and provides 2 reduced nicotinamide adenine dinucleotides (NADH) and 2 adenosine triphosphates (ATP). Pyruvate can be oxidized to acetyl-CoA by pyruvate dehydrogenase (PDH) or converted to oxaloacetic acid by pyruvate carboxylase. In the absence of oxygen, pyruvate is reduced to lactate by lactate dehydrogenase (LDH), or to acetaldehyde by pyruvate decarboxylase (Nishikawa et al., 2014). When the liver is in a pathological state, there is an adaptive metabolic transition from oxidative phosphorylation (OXPHOS), which preferentially produces energy, to glycolysis, in which pyruvate is partially converted to lactic acid (Nishikawa et al., 2014). It was found that glycolysis activity was enhanced and lactic acid levels increased in NAFLD and NASH (Ye et al., 2016). Some glycolytic enzyme levels are elevated in cirrhotic precancerous lesions and are associated with an increase in hepatocellular carcinoma (HCC) (Lee et al., 2018). NAFLD is considered a new major risk factor for HCC, and the increase in serum BCAA levels in NAFLD patients affects glucose metabolism through mTOR signaling (Chalasani et al., 2018; Gaggini et al., 2018; Chakravarthy and Neuschwander-Tetri, 2020). In addition, studies have shown that aerobic glycolysis exists in HCC, and the degree of aerobic glycolysis of cancer cells in HCC is enhanced, and cancer cells preferentially metabolize glucose into lactic acid even under aerobic conditions (Li et al., 2017b). These findings suggested that abnormal glycolysis might promote the development of liver disease.

Traditional cancer treatment methods, such as surgery, chemoradiotherapy and immunotherapy, bring heavy psychological and physical pressure and financial burden to patients [Zhao et al. (2015)]. Studies have found that natural products inhibit glycolysis process and disrupt the proliferation and invasion of cancer by targeting glycolytic or metabolic phenotype [Zhao et al. (2015)]. Furthermore, there are currently no clinically approved drugs to treat NAFLD, which is mainly treated with lifestyle changes through diet and exercise. However, people with NAFLD often have difficulty maintaining an improved lifestyle. Therefore, it is of great practical significance to strengthen the research on the pathogenesis of NAFLD and find safe and effective drugs to prevent and treat NAFLD (Guo et al., 2022a). Many studies have shown that compared with traditional therapies, natural products have obvious advantages in terms of fewer side effects, low toxicity, and light economic burden (Efferth et al., 2007; Hsiao and Liu, 2010).

This paper reviews the important factors and related mechanisms affecting glycolysis in liver diseases. We discuss the role of key glycolytic enzymes and proteins, including hexokinase (HK), phosphofructokinase (PFK), pyruvate kinase (PK), lactate dehydrogenase (LDH), glucose transporters (GLUTs), in liver disease. We also reviewed the role of relevant signaling pathways, including phosphoinositide 3-kinase (PI3K), Wnt/β-catenin, adenosine monophosphate-activated protein kinase (AMPK), in liver disease. Finally, we discuss the role of oncogenes, including c-MYC, HIF-1α, and ROS, in liver disease. We also review the effects of natural products and their active ingredients on metabolic reprogramming in liver disease. Through this review, we hope to identify a number of metabolites with ideal efficacy and low side effects from natural products, and to provide promising therapeutic drugs for metabolic reprogramming of various liver diseases.

### 1.1 NAFLD and glycolysis

**FIGURE 1:**
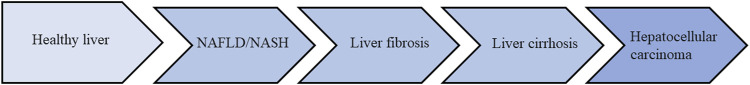
The different stages of development of liver disease.

When the liver is in a pathological state, particularly in their ability to process excess fatty acids via OXPHOS) the resulting mitochondrial dysfunction due to elevated ROS is a main cause of liver dysfunction, which forces hepatocytes to rely on glycolysis as an alternative energy source. Energy metabolism preferentially switches from OXPHOS to glycolysis, with the result that part of the pyruvate is converted to lactate (Nishikawa et al., 2014). NAFLD is driven by inflammatory processes, oxidative stress and insulin resistance triggered by multiple pathways, including NASH, which can lead to liver fibrosis, cirrhosis and HCC (Kanda et al., 2020; Alshehade et al., 2022). The different stages of development of liver disease (Figure 1). Studies have shown that the mechanism of altered glycolysis processes can promote the progression of NAFLD to NASH, and eventually to cirrhosis and HCC (Go et al., 2016). Liver macrophages, neutrophils, dendritic cells, and NK cells are all involved in the development of NASH (Nati et al., 2016). Moreover, macrophages, including Kupffer cells and infiltrating monocytes, play an important role in the progression of NASH. Annexin A5 regulates liver macrophages through interaction with PKM2, improving steatosis, inflammation, and fibrosis in NASH mice (Xu et al., 2020). The activation of glycolysis in Kupffer cells during NASH is induced in part by inhibition of PKM2 upregulation by miR-122-5p (Inomata et al., 2022). Some metabolic pathways involved in glycolysis may be potential therapeutic targets for NAFLD. It was found that the expression of key glycolytic enzyme HK2 was increased in the liver of mice fed high-fat diet (HFD). HFD increased glycolysis by down-regulating the expression of geranylgeranyl diphosphate synthase (GGPPS), accelerating the NAFLD fibrosis process (Liu et al., 2018). Studies have shown that down-regulating NOD-like receptor (NLR) X1 (NLRX1) can inhibit glycolysis, enhance fat oxidation and reduce hepatic steatosis (Kors et al., 2018). Therefore, NLRX1 may be an attractive new therapeutic target for NAFLD and metabolic syndrome. A study has found that hyperacetylation of LDHB is associated with lactic acid accumulation in the liver of NAFLD and NASH in humans and mice. p300/CBP-associated factor (PCAF) mediated acetylation of LDHB K82 significantly decreases LDHB activity, affected lactate clearance in the liver, leading to lactate accumulation. In HFD-induced NASH, acetylated LDHB induces lactic acid accumulation by activating histone hyperacetylation, which intensifies lipid deposition and inflammatory responses (Wang T. et al., 2021). Therefore, targeting the glycolytic pathway may be a treatment option for NAFLD.

### 1.2 Natural products regulate glycolysis in NAFLD

Natural products regulate glycolysis in NAFLD (Table 1). HFD caused a significant decrease in levels of anaerobic (lactic acid) and aerobic glycolytic metabolites (pyruvate) as well as an increase in blood sugar and insulin levels (Chao et al., 2014). Gallic acid (GA) is a natural plant phenolic metabolite isolated from *Cornus officinalis*. It is found in vegetables, tea, grapes, berries and wine and has anti-inflammatory, anti-oxidant and other therapeutic effects (Tanaka et al., 2018). Levels of metabolites associated with anaerobic (lactic acid) and aerobic glycolysis, such as pyruvate and lactic acid, recovered significantly in the GA treatment group. The results indicated that GA had a protective effect on the liver of NAFLD mice, which was partly achieved by improving glycolysis (Chao et al., 2014). Antrodan (Ant), a β-glucan purified from *Antrodia cinnamomea*, has many functions, including anti-cancer, liver protection, and anti-inflammatory effects (Chen et al., 2007; Chiu et al., 2013; Ker et al., 2014; Fa et al., 2015). Ant effectively inhibited glucose and insulin levels, and effectively alleviated glucose metabolism abnormalities in NAFLD through adenosine monophosphate-activated protein kinase (AMPK)/Sirt1/SREBP-1c/PPARγ pathway (Chyau et al., 2020). Vine tea (VT), a tea traditionally used in Chinese botanical drugs, which is derived from *Ampelopsis grossedentata*. It is rich in the natural anti-oxidant dihydromyricetin (ampelopsin). In addition to its many health benefits, rattan tea extract is considered to be a potential natural anti-oxidant (Carneiro et al., 2020). VT decreased serum glucose, decreased the area under the glucose curve in insulin tolerance tests, and decreased fructose-1, 6-phosphate (F1,6P), glucose-6-phosphate (G6P), 6-phospho-gluconate (6PG). Moreover, with low levels of the important intermediates of the phosphor gluconate pathway, pyruvate, G6P, and fructose 6-phosphate (F6P), VT improved HFD-induced glucose metabolism disorders (Wan et al., 2017). *Caralluma fimbriata* (Family: Apocynaceae; CFE), is one such medicinal plant that is becoming increasingly popular, with a variety of bioactive ingredients and properties such as anti-oxidants, liver protection and cancer prevention (Anwar et al., 2022). Extract of CFE promoted the recovery of liver glycolysis (HK, PK, PFK) in HFD rats, suggesting that combined administration of CFE/Met partially corrected the deficiency of glycolysis caused by HFD diet in rats (Gujjala et al., 2017).

**TABLE 1:** Natural products regulate glycolysis in NAFLD.

Name	Origin	Regulatory mechanism	Dose	Cell line/Experiment	References
Gallic acid	*Cornus officinalis*	Increased levels of pyruvate and lactic acid	50 and 100 mg/kg	HFD fed mice	Chao et al. (2014)
Antrodan	*A. cinnamomea*	Inhibition of glucose and insulin levels via AMPK/Sirt1/SREBP-1c/PPARγ pathway	20 and 40 mg/kg	HFD fed mice	Chyau et al. (2020)
Vine tea	*Ampelopsis grossedentata*	Inhibition of F1,6P, G6P,6PG, F6P, pyruvate	500 and 2000 mg/L	HFD fed rats	Wan et al. (2017)
Extract of Caralluma fimbriata	*Caralluma fimbriata*	Increased levels of HK, PK, PFK	200 mg/kg	HFD fed rats	Gujjala et al. (2017)
Saskatoon berry	*Amelanchier alnifolia* Nutt	Increased levels of HK1	8 mg/kg	HFD fed rats	(du Preez et al., 2020)

Saskatoon berry (*Amelanchier alnifolia* Nutt.) is a potential functional food containing phenolic acids, anthocyanins, ellagtannins and flavonols (Nile and Park, 2014; Lachowicz et al., 2017). Saskatoon berry treatment normalized the expression of HK1 and glycogen phosphorylase in liver and increased the expression of G6Pase. These results suggested that saskatoon berry regulates glycolysis, gluconeogenesis, and glycogenesis, improving metabolic syndrome (du Preez et al., 2020).

In summary, most of the experimental studies on the improvement of NAFLD by natural products through glycolysis are mostly studies on complex natural products, which will cause certain uncertainty to the results of the studies. Future studies should identify active natural products and conduct individual natural product studies. In addition, the research on the mechanism of action is not deep enough, and it is necessary to carry out research on the pharmacological action targets of natural products to improve NAFLD on the existing basis, to provide theoretical support for the development of drugs with clear curative effects and clear targets.

### 1.3 Liver fibrosis and glycolysis

Fibrosis is the result of advanced liver damage, it is also closely associated with cirrhosis and liver cancer (Vilar-Gomez et al., 2018). Therefore, the improvement of liver fibrosis has become an important indicator to evaluate the efficacy of NAFLD drugs (Heyens et al., 2021). Moreover, chronic inflammatory environment and fibrotic deposition play a key role in the pathogenesis of HCC (Zhang et al., 2020). However, current drugs to treat liver fibrosis have limited effectiveness and it is important to develop drugs to prevent and reverse fibrosis (Schuppan et al., 2018). Liver fibrosis is characterized by the activation, proliferation, and migration of hepatic stellate cell (HSC) (Jiang et al., 2021). Activated HSCS further promote the formation of excess collagen and the accumulation of extracellular matrix (ECM), leading to persistent chronic liver injury. Without timely intervention, which gradually worsens to cirrhosis and eventually liver cancer (Shan et al., 2019). Activated macrophages release various cytokines, directly damage liver parenchymal cells, promote inflammatory cell infiltration, and activate HSC (Tacke and Zimmermann, 2014). Macrophages follistatin-like protein 1 (FSTL1) binds to PKM2, induces M1 polarization and inflammation, and promotes the progression of liver fibrosis (Rao et al., 2022). Tetramerization of PKM2 can reverse liver fibrosis, and inducing tetramerization of PKM2 to reduce the level of PKM2 dimer may be a potential therapeutic strategy for liver fibrosis (Satyanarayana et al., 2021). It has been found that the expression of GLUT1 and PKM2 is upregulated in liver fibrosis. And the expression of GLUT1 and PKM2 is significantly increased in activated HSC exosomes, suggesting that the exosomes released by HSC are related to HSC activation and glucose uptake (Wan et al., 2019). Activated HSC exosomes affect the metabolism of liver non-parenchymal cells through the transfer of glycolytic-related proteins (Wan et al., 2019). TGF-β1 was found to promote the development of liver fibrosis in mice by activating the Smad, p38 MAPK and PI3K/AKT signaling pathways, causing an increase in aerobic glycolysis in HSC and inducing GLUT1 expression in HSC. After GLUT1 inhibitors were administered, liver inflammation and the degree of liver fibrosis were significantly reduced in mice with liver fibrosis (Zhou et al., 2021). Focal adhesion kinase (FAK) promoted the aerobic glycolysis of cancer cells and fibroblasts, while FAK-related non-kinase kinase (FRNK) inhibited the aerobic glycolysis of HSC by inhibiting the FAK/Ras/c-MYC/ENO1 pathway, thereby improving liver fibrosis. FRNK might be a potential target for treatment of liver fibrosis (Huang et al., 2022). HSC activation is the core process of liver fibrosis, and glycolysis is one of its metabolic markers. Therefore, blocking glycolysis may become a new treatment option for liver fibrosis.

### 1.4 Natural products regulate glycolysis in liver fibrosis

In recent years, many studies have shown that natural products and their active ingredients have anti-fibrosis effects (Table 2). HSC activation was the central event of liver fibrosis. Costunolide is a natural sesquiterpene lactone extracted from *Radix Aucklandiae* and exhibits a variety of biological activities, including anti-fibrotic, anti-oxidant, anti-inflammatory, and anti-cancer properties (Tian et al., 2022; Saraswati et al., 2018; Niu et al., 2021). Costunolide reduced HSC activity by inhibiting the expression of two key markers of HSC activation, a-smooth muscle actin (a-SMA) and Collagen alpha-1(I) chain (COL1A1). It also reduced glucose uptake and consumption, and reduced the level of lactic acid and inhibited HK2 expression and activity, inhibiting glycolysis (Ban et al., 2019). Deoxyelephantopin is a sesquiterpene lactone extracted from Compositae *Elephantopus scaber* L., which has good anti-oxidant and anti-carcinogenic properties (Mehmood et al., 2017). Deoxyelephantopin decreased the expression of a-SMA and α1(I) procollagen II (pro-COL1A1) and inhibited liver fibrosis. In addition, it decreased the expression of HK, PFK2, GLUT4 through the hedgehog pathway, and reduced the production of lactic acid in HSC, inhibiting the production of aerobic glycolysis in HSC (Gao et al., 2019). Curcumin is a bright yellow metabolite isolated from *Curcuma longa* L. (turmeric) plants and has a variety of therapeutic applications, showing liver protection, anti-cancer, anti-inflammatory, anti-oxidant, anti-proliferation effects (Tagde et al., 2021). Curcumin inhibited glycolysis in HSC by decreasing the expression of HK, PFK2, and GLUT4, and lactic acid depending on AMPK activation. Curcumin inhibited the expression of α-SMA and pro-COL1A1, and inhibited liver fibrosis (Lian et al., 2016).

**TABLE 2:** Natural products regulate glycolysis in liver fibrosis.

Name	Origin	Regulatory mechanism	Dose	Cell line/Experiment	References
Costunolide	*Radix Aucklandiae*	Inhibition of HK2	10, 20 and 30 μM	Primary HSCs were isolated from rats	Ban et al. (2019)
Deoxyelephantopin	*Elephantopus scaber* L	Inhibition of HK, PFK2, GLUT4 levels via hedgehog pathway	2.5, 5 and 10 μM	Primary rat HSCs (HSC-T6)	Gao et al. (2019)
Curcumin	*Curcuma longa* L	Inhibition of HK, PFK2, GLUT4 levels via AMPK pathway	20 μM	Primary rat HSCs	Lian et al. (2016)

In conclusion, there are few researches on glycolytic treatment of liver fibrosis based on natural products, and the content of the research is not deep enough. The signaling pathways closely related to liver fibrosis should be associated with glycolysis, such as TGF-β, PDGF, Wnt/β-catenin and Hedgehog signaling pathways to increase the depth of glycolytic treatment of liver fibrosis. In addition, most studies mainly focus on the cellular level, these cannot completely simulate the pathological characteristics of human liver fibrosis, and its drug activity needs to be further studied and confirmed. At present, there is still a lack of specific anti-fibrosis drugs in clinical practice, which leads to no control drugs in animal, cell and clinical experiments, making the natural drug anti-fibrosis research lack of unified and clear efficacy standards. In addition, there are many causes of liver fibrosis, including viral hepatitis, non-alcoholic steatohepatitis and cholestatic liver disease, so it is more meaningful to study cell models and animal models based on different etiology.

### 1.5 Liver cancer and glycolysis

Normal cells produce ATP mainly through OXPHOS under aerobic conditions. In the absence of oxygen, ATP is produced mainly by glycolysis. In the process of rapid proliferation of tumor cells, the demand for energy increases, resulting in a reprogramming process of tumor cell metabolism. Therefore, cancer cells produce ATP mainly through glycolysis, even under aerobic conditions, which is called aerobic glycolysis and also known as the Warburg effect (Koppenol et al., 2011). Tumor cells eventually metabolize glucose into lactic acid through glycolysis, a process that produces energy, but the energy produced by this pathway is much lower than the energy produced by each cycle of tricarboxylic acid. In order to obtain high efficiency glycolysis, tumor cells increase glucose transporters or various key enzymes to promote the efficient entry of nutrients into cells and participate in metabolism (Reckzeh et al., 2019).

The basic way to regulate glycolysis is to change the activity of key glycolytic enzymes, including HK, PFK1 and PK, whose activity directly affects the speed and direction of the entire metabolic pathway (Cui et al., 2022). HK as an important glycolytic enzyme, HKs is responsible for the first rate-limiting step in glucose metabolism, the phosphorylation of glucose to G6P (Tan and Miyamoto, 2015). Currently, there are four different types of HK, namely, HK1-4 (Perrin-Cocon et al., 2018). Among them, HK1 and HK2 are located mainly on the outer membrane of mitochondria, HK3 is located in the perinuclear compartment, and HK4 is located in the cytoplasm. Localization in the mitochondrial outer membrane gives HK2 the advantage of escaping product inhibition and preferentially obtaining mitochondrial ATP (Pedersen, 2008). Among these different HKs, HK2 was upregulated in a variety of cancers and played a key role in the development of Warburg (Xu et al., 2017). Therefore, based on the key role of HK in HCC, HK2 may become a target for the development of new therapies for liver cancer. PFK1 is the second rate-limiting enzyme involved in glycolysis, whose activity is regulated by phosphofructokinase-2/ructose-2, 6-diphosphatase 3 (PFKFB3) (Zuo et al., 2021). PFKFB3 does not directly participate in the catalytic process of glycolysis, but instead produces fructose 2, 6-diphosphate by catalyzing F6P. Fructose 2, 6-diphosphate is an allosteric activator of PFK1 and can significantly enhance the catalytic activity of PFK-1 (Boutard et al., 2019). It was found that the combination of aspirin and sorafenib after inhibiting the expression of PFKFB3 can overcome the resistance of sorafenib by inducing apoptosis of HCC cells, so as to enhance the therapeutic effect of HCC (Li et al., 2017a). Therefore, PFKFB3 is also the key to regulate glycolysis and is another important target for tumor therapy. Another rate-limiting enzyme in glycolysis is PK, which has 4 subtypes, including PKL, PKR, PKM1, and PKM2 (Dong et al., 2016). PKM2 was highly expressed in liver cancer and was associated with poor prognosis (Li et al., 2020). PKM2 existed in different forms, mainly in the form of low activity dimer, which promoted tumor growth. Studies have shown that PKM2 drives HCC progression by inducing immunosuppressive microenvironment and upregulation of PD-L1. Overexpression of PKM2 makes HCC sensitive to immune checkpoint blocking, thereby enhancing IFN-γ-positive CD8 T cells in mouse models of liver cancer (Li et al., 2020). These results indicated that PKM2 was expected to be another therapeutic target in the treatment of liver cancer. In addition, LDH catalyzes the final step in the glycolysis process and is responsible for the mutual conversion of lactic acid and pyruvate (Sharma et al., 2022). There are three subtypes of LDH, including LDHA, LDHB and LDHC, of which LDHA is responsible for converting pyruvate into lactic acid (Feng et al., 2018) and LDHB is responsible for converting lactic acid into pyruvate (Urbańska and Orzechowski, 2019). LDHA was mainly expressed in tumor cells. The expression of LDHA was increased in liver cancer, and a large amount of energy wasrapidly generated through glycolysis to support cancer cell growth (Feng et al., 2018). In addition, it is methylated at R112 and is essential for PRMT3-induced glycolysis and HCC growth (Lei et al., 2022). Therefore, LDHA is a promising therapeutic target. Regulation of glycolysis is also regulated by GLUTs. Of the 14 members of the GLUTs family, only GLUT1-5 is currently the most intensively studied, and they all act as glucose and/or fructose transporters in a variety of tissues and cell types (Pyla et al., 2013; Cui et al., 2022). Hypoxia induced abnormal activation of hypoxia-inducible factor-1α (HIF-1α) in the immune microenvironment, and also upregulated LDHA and GLUT1 to cause glycolysis, which promoted the progression of HCC and led to enhanced drug resistance of cancer cells (Zhou et al., 2022). Therefore, various key enzymes and transporters of glycolysis are expected to become drug targets for liver cancer treatment.

Lactate transport from the extracellular depends on monocarboxylate transporters, MCT family currently consists of 14 members, glycolysis speed is fast, may lead to the increase of lactate production in cancer cells, affect the development and proliferation of tumor cells (Halestrap, 2012). If cancer cells are unable to process lactic acid, it may lead to tumor cell death (Cui et al., 2022). Studies have shown that MCT1 and MCT4 are the main transporters of lactic acid excretion in tumor cells (Ruzzolini et al., 2020; Soni et al., 2020), therefore, MCT may become the target of liver cancer treatment.

Glycolysis also affects the tumor immune microenvironment. The tumor microenvironment is a complex cellular environment. Inducing differentiation of naive CD8^+^T cells is the main anti-tumor mechanism of immune cells. Naive CD8^+^T cells are usually present in a quiescent state. Maintenance of this state is mediated primarily by two molecules: sphingosine 1-phosphate (S1P) and interleukin (IL)-7 (Goronzy et al., 2015; Mendoza et al., 2017). S1P is important for OXPHOS (Mendoza et al., 2017), while IL-7 mainly promotes glucose uptake by GLUT-1, affecting the glycolysis process (Niu et al., 2023). Glycolysis is primarily used to activate T cells, but OXPHOS are also important, and their absence prevents T cell proliferation (Tarasenko et al., 2017). In addition, high levels of lactic acid can cause macrophages to develop into M2 macrophages, inhibit T cell activation and proliferation, and exert their immunosuppressive function by expressing arginase 1 (ARG1) protein (Andrejeva and Rathmell, 2017). Macrophages that ingested glucose can develop into M1 macrophages after receiving interferon gamma (IFN-γ) secreted by Th1 cells, and the anti-tumor ability of M1-macrophages can be enhanced (Andrejeva and Rathmell, 2017). Activation of NK cells require a shift in their metabolism from mitochondrial oxidation to glycolysis (Assmann et al., 2017).

### 1.6 Natural products regulate glycolysis in liver cancer

#### 1.6.1 Targeting glycolytic enzymes

**FIGURE 2:**
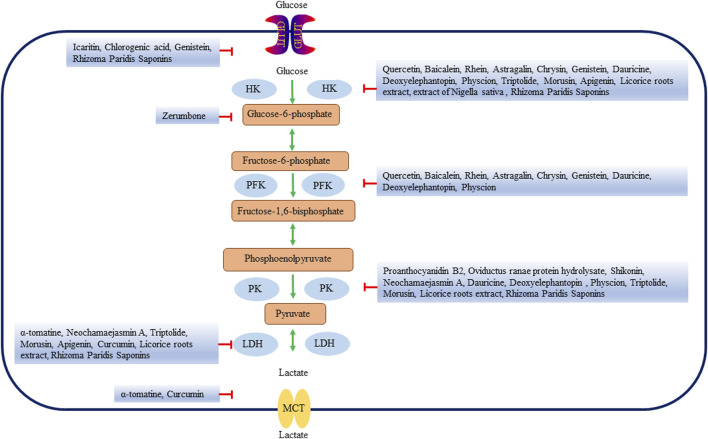
Natural products affect the glycolysis of liver cancer by directly regulating glycolysis enzymes. HK, hexokinase; PFK, Phosphofructokinase; PK, pyruvate kinase; LDH, lactate dehydrogenase; GLUT, glucose transporters; MCTs, Monocarboxylate transporters.

Many natural products affect glycolysis by directly or indirectly regulating key enzymes of glycolysis (Table 3; Figure 2). Quercetin is a flavonoid that is present in a variety of vegetables and fruits (Di Petrillo et al., 2022) and has anti-inflammatory, anti-oxidant and anti-cancer effects (Xu et al., 2019; Saeedi-Boroujeni and Mahmoudian-Sani, 2021; Wang et al., 2022c). Studies have shown that *in vivo* and *in vitro* experiments, quercetin decreases the expression of HK2 in HCC cells (Wu et al., 2019a). Baicalein, one of the main bioactive metabolites isolated from *Scutellaria baicalensis,* has anti-inflammatory, anti-lipogenesis, anti-viral and cardiovascular protective effects (He et al., 2021). Baicalein inhibited the activity and energy metabolism of liver cancer cells. Baicalein also significantly inhibited the expression of HK2 and decreased the glycolysis ability of liver cancer cells (郭舜 et al., 2021). In glycolytic-dependent cells, the binding of HK and VDAC was an important factor in the maintenance of glycolysis and could inhibit mitochondrial energy metabolism (Todisco et al., 2016). Rhein is an anthraquinone metabolite extracted from the Traditional Chinese Medicine (TCM) rhubarb [Huang et al. (2022)], which has anti-cancer, anti-inflammatory and anti-viral effects (Wang et al., 2018; Zhuang et al., 2019; Bu et al., 2020). Rhein dissociated the binding of VDAC and HK, inhibited glycolysis, reduced ATP in liver cancer, inducing apoptosis of liver cancer cells (Wu et al., 2019b). Oviductus Ranae, derived from the dried tubular product of *Rana temporaria chensinensis* David, has anti-fatigue and increased immune biological activity (Xiao et al., 2019). Oviductus Ranae is a precious natural product in northeast China, which has been developed into a series of health food and TCM (Wang et al., 2021a). Oviductus ranae protein hydrolysate (ORPH) treatment decreased the expression of PKM2 by upregulating miR-491-5p in a post-transcriptional manner, and inhibited the growth, metastasis and glycolysis of mouse liver cancer cells (Xu et al., 2018). Astragalin (ASG) is a flavonoid that was found in a variety of botanical drugs, such as *Radix astragali*, *Morus alba* and *Cassia alata*, as well as in some fruits and vegetables (Liu et al., 2019). ASG has been widely used in various pharmacological fields because of its anti-inflammatory, anti-oxidant and inhibitory effect on malignant tumor cells (Harikrishnan et al., 2020). ASG could inhibit HCC cell proliferation by promoting microRNA-125b (miR-125b) and metabolic reprogramming, reducing HK2 expression and inhibiting glycolysis in HCC cells (Li et al., 2017c). Chrysin is a bioactive flavonoid derived from plant extracts, found in blue passion flower, propolis and honey, and is widely used as a Chinese herbal medicine in China. Chrysin not only has anti-oxidant, anti-inflammatory and other biological activities, but also has anti-cancer effect (Yao et al., 2014; Zhang et al., 2015; Tang et al., 2016). Chrysin or its derivatives significantly inhibited glucose uptake and lactate production in HCC cells by decreasing HK2 expression. Reduced expression of HK2 bond to voltage-dependent anion channel 1 on mitochondria, leading to transport of Bcl-2-associated X protein (Bax) from cytoplasm to mitochondria and inducing apoptosis (Xu et al., 2017). Shikonin is a naturally occurring naphthoquinone isolated from the root of the plant *Lithospermum erythrorhizon*. Studies have shown that comfrey and its derivatives have anti-cancer effects on many types of tumors (Lee et al., 2014; Ruan et al., 2021). By inhibiting PKM2, Shikonin decreased the expression of cyclinD1, inhibited liver cancer glycolysis and cell proliferation, and induced cell apoptosis. The effect of Shikonin on the proliferation, apoptosis and glycolysis of HCC cells will make it a promising drug for the treatment of HCC (Liu et al., 2020). Icaritin is an active component of Chinese botanical drug *Epimedium*. It has a wide range of biological and pharmacological functions, including anti-oxidant and anti-cancer (He et al., 2010; Zhou et al., 2011; Qin et al., 2020). Icaritin induced upregulation of FAM99A expression in HCC cells, blocked JAK2/STAT3 pathway, and inhibited GLUT1-mediated glycolysis and HCC cell viability (Zheng et al., 2021). Chlorogenic acid (CGA) is a dietary phenolic acid produced by a variety of plants, such as *Sonchus oleraceus Linn*. CGA is the most prevalent metabolite in the phenolic acid group, which is also found in tea and coffee extracts (Gupta et al., 2022). CGA has a wide range of effects, such as anti-cancer, anti-bacterial, anti-oxidant and so on (Zeng et al., 2021). The hepatoprotective effect of CGA might be related to increasing the production of ATP, stimulating mitochondrial OXPHOS and inhibiting glycolysis (Zhou et al., 2016). CGA also prevented the glucose-induced decline in GLUT4 levels and regulated glucose uptake and transport in HepG2 cells (Chen et al., 2019). Oleuropein is an iridoid phenolic metabolite composed of three structural subunits: hydroxytyrosol, enolic acid and glucose molecules. It is also reported to be a chemical classification marker for olives. Oleuropein has been reported to have a variety of biological activities, including anti-dyslipidemia, anti-atherosclerosis, anti-inflammatory, anti-diabetes, and liver protective effects (Ahamad et al., 2019). Glucose-6-phosphate isomerase (GPI) was a key enzyme in glycolysis. The expression of GPI in tumor cells affected different physiological functions and signal transduction. GPI was the direct target of oleuropein, which could inhibit liver cancer glycolysis by inhibiting GPI, and it showed good anti-tumor activity *in vivo* without adverse side effects (Hong et al., 2023). Erianin, extracted from the rare Chinese medicine *Dendrobium chrysotoxum Lindl*, is a small molecule natural metabolite with a wide range of anti-cancer potential *in vivo and in vitro* (Sun et al., 2020). Erianin effectively inhibited the enzyme activity of pyruvate carboxylase (PC), promoted mitochondrial oxidative stress, inhibited glycolysis, inducing insufficient energy required for the proliferation of liver cancer cells (Sheng et al., 2022). Zerumbone is a natural metabolite of the ginger plant *Zingiber zerumbet* (L.) Smith, which is used to treat a wide variety of ailments. Zerumbone’s anti-cancer properties have been reported *in vitro* and *in vivo* studies of a variety of cancers (Zainal et al., 2018; Girisa et al., 2019). Zerumbone blocked the binding of G6P through the pentose phosphate pathway, reduced glucose consumption and lactic acid production, inhibited glycolysis, inducing cell cycle arrest and apoptosis of liver cancer cells (Wani et al., 2018).

**TABLE 3:** Natural products regulate glycolysis via glycolytic enzymes in liver cancer.

Name	Origin	Target glycolytic enzymes	Regulatory mechanism	Dose	Cell line/Experiment	References
Quercetin	Vegetables and fruits	HK2	downregulate	12.5,25,50 μM and 50 mg/kg	SMMG-7721, BEL-7402 cells and BALB/c nude mice	Wu et al. (2019a)	
Baicalein	*Scutellaria baicalensis*	HK2	downregulate	5,10 and 20 μM	SMMC-7721 and HepG2 cells	(郭舜 et al., 2021)	
Rhein	rhubarb	HK2	downregulate	5,10,20,40,60,80 μM	SMMC-7721 and SMMC-7721/DOX cells	Wu et al. (2019b)	
Proanthocyanidin B2	Vegetables and fruits	PKM2	downregulate	10,20,40,60,80,100,120 and 140 μM	HCC-LM3, SMMC-7721, Bel-7402, Huh-7 and HepG2 cells	Feng et al. (2019)	
Oviductus ranae protein hydrolysate	*Rana temporaria chensinensis* David	PKM2	downregulate	400 ug/mL for HepG2 cells and 450 ug/mL for Hep3B cells	Hep3B, HepG2 cells and clinical samples	Xu et al. (2018)	
Astragalin	Botanical drugs, vegetables and fruits	HK2	downregulate	11,33 μM and 10,20 mg/kg	HepG2, Huh-7, HL-7702, H22 cells and Kunming mice, athymic nude mice	Li et al. (2017c)	
Chrysin	Botanical drugs	HK2	downregulate	15,30,60 μM and 30 mg/kg	HepG2, Hep3B, Huh-7, HCC-LM3, Bel-7402, SMMC-7721 and nu/nu athymic nude mice	Xu et al. (2017)	
Shikonin	*Lithospermum erythrorhizon*	PKM2	downregulate	1,2,3 μM and 5 mg/kg	HCC-LM3, SMMC-7721, Huh-7, HepG2 cells and BALB/c nude mice	Liu et al. (2020)	
Icaritin	*Epimedium*	GLUT1	downregulate	2.5,5 and 10 μM	HepG2, HCC-LM3 cells and nude mice	Zheng et al. (2021)	
Chlorogenic acid	Plants	GLUT4	downregulate	25,50 and 100 μg/mL	HepG2 cells	Chen et al. (2019)	
Oleuropein	Olives	GPI	downregulate	1,10,25,100,250 μM and 200 mg/kg	HepG2, HuH-7 cells and Balb/c mouse	Hong et al. (2023)	
Erianin	*Dendrobium chrysotoxum Lindl*	PC	downregulate	10,20,30,40,50,100,200 and 400 nM	HepG2, MHCC97, SK-Hep-1 and HCC-LM3 cells	Sheng et al. (2022)	
Zerumbone	*Zingiber zerumbet* (L.) Smith	Glucose-6-phosphate	downregulate	50,100,150,200 µM and 20 mg/kg	HepG2, Hep3B, Sk-Hep-1, SNU-182, SNU-449, HCC-LM3 cells and NSG mice	Wani et al. (2018)	
α-tomatine	Tomato	LDHA, MCT4	downregulate	0.5,1,15,2 and 2.5 µM	HuH-7 cells	(何志龙 et al., 2022)	
Neochamaejasmin A	*Stellera chamaejasme*	PK, LDH	downregulate	10,20,40,80 μg/mL	HepG2 cells	(丁杨芳 et al., 2019)	
Genistein	Soybeans	HK2, GLUT1	downregulate	20,40,60,80,100,140 µM and 20,40,80 mg/kg	HCC-LM3, SMMC-7721, Hep3B, Bel-7402, Huh-7 cells and athymic BALB/c nu/nu mice	Li et al. (2017b)	
Dauricine	*Menispermum dauricum* DC.	HK2, PKM2	downregulate	2 μg/mL and 10 mg/kg	HepG2, Huh-7, Hep3B and athymic nude mice	Li et al. (2018)	
Deoxyelephantopin	*Elephantopus scaber* L	HK2, PFK1, PKM2	downregulate	0.625,1.25,2.5,5,10,20,40 and 80 µM	HepG2 cells	(吴红雁 et al., 2023)	
Physcion	*Rheum officinale* Baill	HK2, PFKFB3, PKM2	downregulate	1.25,2.5,5,10,20 and 40 µM	HepG2 cells	(陶正娣 et al., 2022)	
Triptolide	*Tripterygium wilfordii Hook f*	HK2, PKM2, LDHA	downregulate	10,40,60 and 100 nM	SMMC-7721 cells	(李恬 et al., 2020)	
Morusin	*Morus alba*	HK2, PKM2, LDH	downregulate	2.5,5,10,20 and 40 μM	Huh7 and Hep3B cells	Cho et al. (2021)	
Apigenin	Vegetables and fruits	HK2, LDHA, PDHK1	downregulate	20,40,80 μM and 400 mg/kg	HepG2 cells and athymic nude mice	(张睿 et al., 2020)	
Curcumin	*Curcuma longa* L	LDHA, MCT1	downregulate	1,2,5,10 μM	HepG2 and HuT78 cells	Soni et al. (2020)	
Licorice roots extract	*Glycyrrhiza glabra* L	HK2, PKM2, LDHA	downregulate	1.562–200 mg/mL	HepG2 cells	Abdel-Wahab et al. (2021)	
Scopolin	*Smilax china* L	GPI, GPD2, PGK2	downregulate	25,50,100,200 μM and 20,50,100 mg/kg	HepG2 cells and BALB/c mice	Wang et al. (2022a)	
Extract of Nigella sativa	Nigella sativa	HK, GAPDH	downregulate	1 g/kg	Wister Albino rats	Abdel-Hamid et al. (2013)	
Rhizoma Paridis Saponins	Rhizoma Paridis	HK2, PKM2, LDHA, GLUT1	downregulate	100 mg/kg	Kunming mice	Qiu et al. (2016)	

Neochamaejasmin A is a major component in the stem root of *Stellera chamaejasme*, which has anti-cancer effects on tumor cells (Liu et al., 2008). Neochamaejasmin A inhibited the proliferation of tumor cells by inhibiting glucose uptake and lactate production in HepG2 cells, and reducing the expression of glycolytic related proteins PK and LDH (丁杨芳 et al., 2019). Genistein is an isoflavone found in soybeans. It plays an important role in the occurrence and development of cancer and the prevention and treatment of common diseases such as metabolic syndrome by inhibiting inflammation and regulating metabolic pathways (Xu et al., 2022). Genistein inhibited aerobic glycolysis by decreasing GLUT1 and HK2 (Li et al., 2017b). Dauricine (Dau), an alkaloid metabolite isolated from the roots of *Menispermum dauricum* DC., inhibits tumor growth. By increasing miR-199a, Dau directly downregulated HK2 and PKM2, inhibited glycolysis and increased OXPHOS, inhibited liver cancer cell proliferation, and sensitized sorafenib therapy (Li et al., 2018).

Physcion extracted from *Rheum officinale* Baill., is a naturally occurring anthraquinone derivative with anti-cancer, anti-bacterial, anti-viral, anti-inflammatory and other biological activities (Zhang et al., 2021). Physcion significantly downregulated the expression of HK2, PFKFB3, PKM2, and inhibited glucose uptake and lactic acid production. These results showed that Physcion inhibited the proliferation of liver cancer cells by interfering with the process of glycolytic energy metabolism (陶正娣 et al., 2022). Triptolide is a natural epoxy diterpenoid metabolite derived from botanical drug *Tripterygium wilfordii Hook f*, which has strong anti-cancer properties (Chen et al., 2018; Cai et al., 2021). Licorice plant, (*Glycyrrhiza glabra* L.), have been widely used in TCM for the treatment of different diseases, for its role in nourishing qi, tonifying spleen and stomach, and harmonizing prescriptions (Abdel-Wahab et al., 2021; Shikov et al., 2022). Licorice roots extract induced apoptosis and cycle arrest of liver cancer cells, inhibited HK2, PKM2, and LDHA enzymes, and inhibited glycolysis by up-regulating multiple tumor suppressor genes miRNAs (Abdel-Wahab et al., 2021). Smilax china L. is a well-known Chinese medicine used as an anti-inflammatory, anti-cancer and analgesic (Yu et al., 2014a; Feng et al., 2020). Scopolin obtained from *Smilax china* L. plays the role of anti-HCC. Scopolin regulated glycolysis-related proteins glucose-6-phosphate isomerase (GPI), glycerol-3-phosphate dehydrogenase, mitochondrial (GPD2) and phosphoglycerate kinase 2 (PGK2) expression and inhibited protein-protein interaction, reduced energy metabolism in liver cancer tissue, inhibiting tumor growth (Wang et al., 2022a). *Nigella sativa* (NS), commonly known as the black seed or black cumin “Al-Habba Al-Sauda”, is the seed of an enveloped plant belonging to the Ranunculaceae family (Amin and Hosseinzadeh, 2016). It has been used as a spice and food preservative, as well as a protective and therapeutic drug for many diseases (Zielińska et al., 2021). Serum HK and GAPDH were increased in liver cancer group. Extract of Nigella sativa (MENS) inhibited glycolysis by reducing the expression of these enzymes and has chemical preventive effects on the progression of liver cancer (Abdel-Hamid et al., 2013). *Rhizoma Paridis Saponins* (RPS), a natural product purified from the commonly used Chinese medicine Rhizoma Paridis, is not only inhibits liver fibrosis and cirrhosis, but also inhibits the growth of a variety of cancers (Man et al., 2014a; Man et al., 2014b). RPS decreased the expression of GLUT1, HK2, PKM2, and LDHA. RPS also reversed aerobic glycolysis by activating tumor suppressor genes p53 and PTEN, and inhibited the proliferation of mouse liver cancer H22 tumors (Qiu et al., 2016). In summary, as a rich resource, natural products show potential as glycolysis inhibitors in the future clinical treatment of liver cancer.

#### 1.6.2 Targeting multiple signaling pathway in liver cancer

**FIGURE 3:**
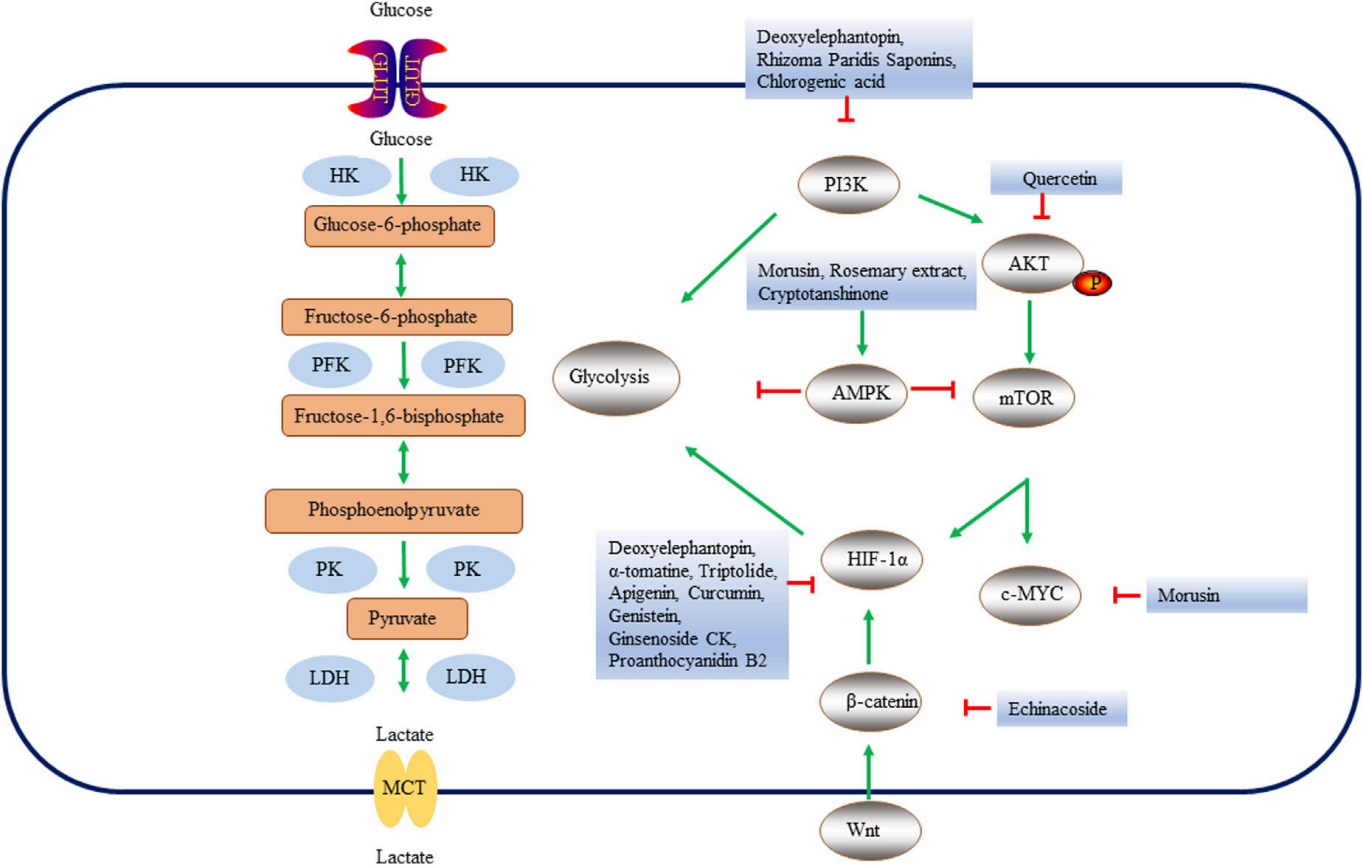
Key enzymes, proteins and pathways of natural products regulating glycolysis process in liver cancer. Natural products regulate glycolysis in three ways. First, natural products affect the glycolysis of liver cancer cells by directly regulating glycolytic enzymes. Secondly, natural products can inhibit liver cancer cell glycolysis through PI3K, Wnt/β-catenin or AMPK pathways. Finally, natural products may regulate genes related to glycolysis by regulating oncogenes c-MYC, HIF-1α, etc., thus changing the metabolic pathway of liver cancer. HK, hexokinase; PFK, Phosphofructokinase; PK, pyruvate kinase; LDH, lactate dehydrogenase; GLUT, glucose transporters; MCTs, Monocarboxylate transporters; HIF-1α, Hypoxia Inducible Factor 1 Subunit Alpha; AMPK, AMP-dependent protein kinase; PI3K, Phosphatidylinositol 3-kinase; AKT, protein kinase B; mTOR, mammalian target of rapamycin.

Various signaling pathways play an important role in the glycolysis of liver cancer (Table 4; Figure 3). Studies have shown that the activated PI3K/AKT signaling pathway stimulates glucose uptake by regulating GLUT1 expression, enhances glycolysis, drives lactic acid production in cancer cells, inhibits macromolecular degradation, and affects tumor cell metabolism (Wasik and Lehtonen, 2018; Jin et al., 2021). Quercetin has been found to reduce HK2 levels and inhibit the AKT/mTOR pathway in liver cancer cells *in vivo* and *in vitro* (Wu et al., 2019a). In addition to its role in liver fibrosis, Deoxyelephantopin also plays an important role in liver cancer. Deoxyelephantopin reduced glucose uptake and lactic acid production and inhibited glycolysis through PI3K/Akt/mTOR/HIF-1α signaling pathway, thus inhibiting the proliferation and migration of HepG2 cells (吴红雁 et al., 2023). It was found that the combination of RPS sorafenib increased the anti-cancer effect, overcoming the tolerance of sorafenib by protecting mitochondrial damage, inhibiting anaerobic glycolytic through PI3K/AKT/mTOR pathway (Yao et al., 2018). CGA regulated glucose uptake and transport in HepG2 cells through the PI3K/AKT pathway (Chen et al., 2019). *Prunella vulgaris* is dried fruit spike of Lamiacea plant *P. vulgaris* L., which is an important medicinal plant mainly found in Europe and Asia (Chang et al., 2023). Prunella vulgaris total flavonoids inhibit the proliferation of liver cancer (Song et al., 2021). The Prunella vulgaris total flavonoids activated Bcl-2/Bax protein to induce apoptosis of liver cancer cells, and the mechanism might be related to the inhibition of aerobic glycolysis and OXPHOS levels of liver cancer cells (宋亚刚 et al., 2020). The Wnt/β-catenin signaling pathway stimulates glycolysis by up-regulating the expression of HK2, LDHA and pyruvate dehydrogenase kinase 1 (PDK1) (Fang et al., 2019). The Wnt/β-catenin pathway stimulates the downstream PI3K/AKT pathway and HIF-1α, thereby indirectly activating aerobic glycolysis (Vallée et al., 2017). Therefore, targeting the Wnt/β-catenin signaling pathway can regulate glycolysis. Echinacoside (ECH) is an active component of Cistanche salsa, which has strong anti-proliferation and pro-apoptotic activities in various cancers including HCC (Ye et al., 2019; Wang et al., 2022b). Studies have shown that ubiquitin protein ligase E3 component N-recognin 5 (UBR5) expression is associated with decreased apoptosis and increased glycolysis of hepatoma cells through β-catenin signaling pathway. AMPG nanocomposites have low cytotoxicity and good biosafety. The ECH of AMPG effectively reduced glycolysis and promoted the apoptosis of liver cancer cells (Wang et al., 2022b). HIF-1α and AMPK signaling pathways are major regulators of glycolysis and OXPHOS and are critical for metabolic reprogramming of tumor cells (Chen et al., 2022). AMPK is a key sensor and regulator of cellular metabolism (González et al., 2020). AMPK is a heterotrimer complex consisting of a catalytic α subunit and two regulatory β and γ subunits (Herzig and Shaw, 2018). Morusin is a kind of naturally existing prenylated flavonoid isolated from the root bark of *M. alba*, which has the effect of inhibiting cancer (Wang et al., 2020). Morusin inhibited HK2, PKM2 and LDH expression and reduced lactic acid, glucose and c-MYC, activated AMPK through AMPK pathway, which played an important role in anti-HCC (Cho et al., 2021). Rosemary extract from the plant *Rosmarinus officinalis* L., has anti-oxidant, anti-cancer, anti-bacterial and other effects (Jaglanian and Tsiani, 2020). Rosemary extract significantly increased glucose consumption in HepG2 cells and promoted liver glycolysis and fatty acid oxidation by activating AMPK and PPAR pathways (Tu et al., 2013). Cryptotanshinone is a liposoluble soluble diterpene derivative mainly found in the genus Salvia, among which *S. miltiorrhiza* Bunge, is a diterpene-rich plant (Dalil et al., 2022). Arsenic trioxide cooperate cryptotanshinone (ACCS) inhibited liver cancer by increasing AMPK phosphorylation and activating AMPK signaling pathway, which enhanced glucose utilization and glycolysis of macrophages (Jiang et al., 2022).

**TABLE 4:** Natural products regulate glycolysis via multiple signaling pathways in liver cancer.

Name	Origin	Regulatory mechanism	Dose	Cell line/Experiment	References
Quercetin	Vegetables and fruits	AKT, mTOR	12.5,25,50 μM and 50 mg/kg	SMMG-7721, BEL-7402 cells and BALB/c nude mice	Wu et al. (2019a)
Deoxyelephantopin	*Elephantopus scaber* L	PI3K, Akt, mTOR	0.625,1.25,2.5,5,10,20,40 and 80 µM	HepG2 cells	(吴红雁 et al., 2023)
Rhizoma Paridis Saponins	Rhizoma Paridis	PI3K, Akt, mTOR	80 mg/kg	Kunming mice	Yao et al. (2018)
Chlorogenic acid	Plants	PI3K, AKT	25,50 and 100 μg/mL	HepG2 cells	Chen et al. (2019)
Prunella vulgaris total flavonoids	*Prunella vulgaris* L	Bcl-2, Bax	50,100,200,400 and 800 μg/μL	SMMG7721 cells	(宋亚刚 et al., 2020)
Echinacoside	*Cistanche salsa*	β-catenin	1,2,5,10 and 20 μg/mL	HepG2 and Huh7 cells and clinical samples	Wang et al. (2022b)
Morusin	*Morus alba*	AMPK, p-mTOR	2.5,5,10,20 and 40 μM	Huh7 and Hep3B cells	Cho et al. (2021)
Rosemary extract	*Rosmarinus officinalis* L	AMPK	2,10 and 50 μg/mL	HepG2 cells	Tu et al. (2013)
Cryptotanshinone	*S. miltiorrhiza* Bunge	AMPK	5,10,15,20,25 μM and 2.5 mg/kg	H22, Hepa1-6 cells and C57BL/6J mice	Jiang et al. (2022)

#### 1.6.3 Targeting multiple oncogenes in liver cancer

Multiple oncogenes play an important role in the glycolysis of liver cancer (Table 5). Oncogenes maintained the survival and development of cancer cells through reprogramming of glycolytic metabolism [Mukhopadhyay et al. (2021)]. Studies have found that abnormal expression of MYC exists in cancer, and the expression of MYC is closely related to genes regulating glucose metabolism, such as GLUT1, HK2, PFKM, etc. (Wokolorczyk et al., 2008; Dang et al., 2009). Cancer cells also used activation of HIF-1α to increase glucose uptake and glycolysis flux, promoted glucose catabolism and adapted to low oxygen environment to ensure tumor growth (Lee et al., 2020). ROS stimulated carcinogenic signaling, specifically HIF-1α. ROS stabilized HIF-1α protein by inhibiting propyl hydroxylase protein D (PHD) (Ren et al., 2022). In recent years, more and more natural products targeting HIF-1α and ROS expression and inhibiting liver cancer glycolysis have been studied for the treatment of liver cancer. Deoxyelephantopin reduced the glucose uptake and lactic acid production of liver cancer cells, downregulated the key glycolysis enzymes HK2, PFK1, and PKM2, inhibited the glycolysis of liver cancer through the PI3K/AKT/mTOR/HIF-1α signaling pathway, inhibiting the proliferation and migration of HepG2 cells (吴红雁 et al., 2023). α-tomatine, a steroid sugar alkaloid, is abundant in the flowers, leaves, calyx and immature fruits of tomato. It has various biological activities, such as anti-cancer, anti-inflammatory and anti-viral (Zhao et al., 2015; Fujimaki et al., 2022). α-tomatine reduced the expression of LDHA and MCT4, inhibited the uptake of glucose, reduced lactic acid and intracellular ATP, reduce the expression of HIF-1α, inhibiting glycolysis, proliferation and metastasis of Huh-7 cells (何志龙 et al., 2022). Triptolide could reduce the expression of HIF-1α, inhibit the production of glucose and lactic acid, inhibiting the glycolysis and proliferation and metastasis of liver cancer cells (李恬 et al., 2020). Apigenin is a natural flavonoid that is found in a variety of natural plants, including most vegetables and fruits and exhibits many beneficial effects, including anti-cancer, anti-oxidant and anti-bacterial (Ginwala et al., 2019; Wang et al., 2021b; Kashyap et al., 2022). Apigenin inhibited HIF-1α in HepG2 cells, and decreased the expression of glycolytic related proteins (HK2, LDHA, PDHK1), thereby inhibiting the anti-cancer effect of glycolysis (张睿 et al., 2020). Curcumin inhibited anaerobic glycolysis by inhibiting the expression of LDH and HIF-1α, which strengthened the anti-HCC effect of sorafenib (Man et al., 2020). Curcumin also reduced the expression of HIF-1α, inhibited glucose consumption and lactic acid production, alleviating the drug resistance of liver cancer cells to chemotherapy (Soni et al., 2020). By directly down-regulating HIF-1α, Genistein made aerobic glycolytic HCC cells sensitive to apoptosis, and thus inactivated GLUT1 and HK2 to inhibit aerobic glycolysis (Li et al., 2017b). Proanthocyanidin B2 (PB2) is widely exists in natural product, such as fruits and vegetables and has strong anti-oxidant activity because the phenolic hydrogen atoms can effectively intercept free radicals in the free radical chain reaction (Snow et al., 2019). The anti-cancer properties of these metabolites have been well documented, and these effects are mainly attributable to their powerful anti-oxidant and anti-inflammatory effects (Al-Ishaq et al., 2020). PB2 inhibited the expression and nuclear translocation of PKM2, thereby disrupting the interaction between PKM2/HSP90/HIF-1α, and inhibiting the aerobic glycolysis and proliferation of liver cancer cells (Feng et al., 2019). Ginseng (Panax ginseng C. A. Meyer, Family Araliaceae) is one of the major medicinal and nutraceutical plants (Murthy et al., 2018). Ginsenoside CK is one of the most abundant intestinal metabolites of ginsenoside prototype saponins (Guo et al., 2020). It has anti-cancer and anti-inflammation effects, among others (Zhang et al., 2013; Li et al., 2014). Ginsenoside CK inhibited the expression of HIF-1α under hypoxia condition, promoted the ubiquitination degradation of HIF-1α, thus inhibiting the glycolysis and proliferation of hepatoma cells (苏杰琳 et al., 2021).

**TABLE 5:** Natural products regulate glycolysis via oncogenes in liver cancer.

Name	Origin	Regulatory mechanism	Dose	Cell line/Experiment	References
Morusin	*Morus alba*	c-MYC	2.5,5,10,20 and 40 μM	Huh7 and Hep3B cells	Cho et al. (2021)
Deoxyelephantopin	*Elephantopus scaber* L	HIF-1α	0.625,1.25,2.5,5,10,20,40 and 80 µM	HepG2 cells	(吴红雁 et al., 2023)
α-tomatine	Tomato	HIF-1α	0.5,1,15,2 and 2.5 µM	HuH-7 cells	(何志龙 et al., 2022)
Triptolide	*Tripterygium wilfordii Hook f*	HIF-1α	10,40,60 and 100 nM	SMMC-7721 cells	(李恬 et al., 2020)
Apigenin	Vegetables and fruits	HIF-1α	20,40,80 μM and 400 mg/kg	HepG2 cells and athymic nude mice	(张睿 et al., 2020)
Curcumin	*Curcuma longa* L	HIF-1α	1,2,5,10 μM	HepG2 and HuT78 cells	Soni et al. (2020)
Genistein	Soybeans	HIF-1α	20,40,60,80,100,140 µM and 20,40,80 mg/kg	HCC-LM3, SMMC-7721, Hep3B, Bel-7402, Huh-7 cells and athymic BALB/c nu/nu mice	Li et al. (2017b)
Proanthocyanidin B2	Vegetables and fruits	HIF-1α	10,20,40,60,80,100,120 and 140 μM	HCC-LM3, SMMC-7721, Bel-7402, Huh-7 and HepG2 cells	Feng et al. (2019)
Ginsenoside CK	Panax ginseng C. A. Meyer	HIF-1α	20,40,60 μM	Bel-7404 cells	(苏杰琳 et al., 2021)
Prunella vulgaris total flavonoids	*Prunella vulgaris* L	ROS	50,100,200,400 and 800 μg/μL	SMMG7721 cells	(宋亚刚 et al., 2020)

At present, there have been many studies on natural products regulating glycolysis in the treatment of liver disease, but most of the studies focused on the changes in glycolysis of liver cancer cells. However, with the in-depth study of liver cancer in recent years, we found that it is far from enough to study single cells of liver cancer cells. Glycolysis products in liver cancer cells also play an important role in the microenvironment of liver cancer. We should also study how glycolysis affects other cell types in the liver cancer microenvironment. For example, glycolysis product lactic acid has a profound effect on macrophage activation and T cell exhaustion. The further study of glycolysis also provides an alternative target for the existing combination therapy of liver cancer.

## 2 Conclusion and future perspectives

In NAFLD, glycolysis is significantly enhanced, resulting in increased levels of pyruvate. Pyruvate is enhanced in NAFLD by conversion to oxaloacetic acid or lactic acid (Wang et al., 2021c). The enhancement of glycolytic activity will promote the production of mitochondrial ROS, leading to the progression of NAFLD to NASH, and inhibiting mtROS to maintain mitochondrial homeostasis may be a potential treatment for NAFLD and prevent the further development of the disease (Shimada et al., 2012). In addition, the 2020 proposal to change NAFLD to metabolic (dysfunction) associated fatty liver disease (MAFLD) puts more emphasis on the importance of metabolism (Eslam et al., 2020). Although the metabolic abnormalities of NAFLD are not only the abnormalities of glycolysis and oxidative phosphorylation, glycolysis plays a more important role in the progression of NAFLD disease. More and more evidences showed that HSC played an important role in the process of liver fibrosis. Glucose metabolic reprogramming played an important role in the activation of HSC, mainly through upregulation of glycolysis to meet the energy requirements of HSC activation (Guo et al., 2022b). Therefore, natural products blocking glycolysis through some metabolic pathways may become a new treatment option for liver fibrosis. The occurrence of various types of liver cancer is closely related to liver fibrosis and cirrhosis, and the abnormal state of glycolysis in liver cancer has also aroused our attention to the changes of glycolysis in liver cirrhosis in the early stage of liver cancer.

Metabolic reprogramming is a core marker of cancer and is crucial for tumorigenesis and progression (Liu et al., 2021). Glycolysis plays an important role in promoting the progression of liver cancer, including proliferation, migration and drug resistance.

Natural products may be important for overcoming limitations in liver cancer treatment by targeting key enzymes contained in glycolysis (such as HK2, PFK, or PKM2) and other signaling pathways. Importantly, natural products inhibit key glycolytic enzymes and proteins, and inhibit oncogenes c-MYC, HIF-1α, and ROS-mediated metabolic reprogramming toward glycolytic phenotypes. Moreover, it has advantages in improving metabolic reprogramming of tumor cells, and in glycolytic signaling pathways, such as PI3K, Wnt/β-catenin and AMPK may be the main targets of natural products. The multi-target and multi-pathway therapeutic effect of natural products on liver disease is its advantage, but the intensity of the effect on the target is not enough. In addition, the rate-limiting enzymes in the process of glycolysis have a variety of isoenzymes, such as HK, HK1-2 plays a role in the glycolysis of liver cancer, but glucokinase (HK4) plays a role in NAFLD. Therefore, the study of natural products should select different enzymes according to different liver diseases (Pusec et al., 2019). The depth of research is more limited to the study of hepatocyte glycolysis abnormalities, and more attention should be paid to the study of non-hepatocyte glycolysis in different liver diseases, such as fibroblasts, Kupffer cells, macrophages and T cells. In addition, many studies on natural products are in the pre-clinical stage, and more clinical data are needed to support the safety and effectiveness of natural products. In addition, the research on the treatment of natural products for liver disease should not only stay in pre-clinical research, but also strive to be transformed into clinical drugs. Since natural products have the potential to treat diseases, they should show their advantages.

The occurrence of liver diseases is closely related to the metabolic dysfunction of liver cells, and the abnormal glycolysis function concerned in this paper is only a part of the metabolic abnormalities, and the abnormal glycolysis function is closely related to and mutually influenced by the abnormal oxidative phosphorylation. Due to the limitation of the length of the study, it is difficult for us to introduce different kinds of metabolic abnormalities in different liver disease at the same time in one article. Therefore, we selected the dysfunction of glycolysis in liver disease to introduce it. Glycolysis is an important part of liver cells to provide energy, and it is also an important metabolic mode of liver cancer. Therefore, we choose glycolysis to review.
